# Two Unusual Cases of Pituitary Tumors Presenting with Pediatric Acromegaly

**DOI:** 10.7759/cureus.31604

**Published:** 2022-11-17

**Authors:** Shilpa Gurnurkar, Unnati Patel, Jennifer Seekford, Mauri Carakushansky, Madhuradhar Chegondi

**Affiliations:** 1 Pediatric Endocrinology, Nemours Children's Hospital, Florida, Orlando, USA; 2 Pediatrics/Critical Care Medicine, Carver College of Medicine, University of Iowa, Iowa City, USA

**Keywords:** pediatric, pituitary adenoma, growth hormone, acromegaly, gigantism

## Abstract

Gigantism and acromegaly are most commonly caused by a growth hormone (GH)-secreting pituitary adenoma. Pediatric cases are diagnostically and therapeutically challenging due to their insidious nature. This article presents two adolescent females who were referred to the endocrinology clinic primarily for the evaluation of menstrual disorders rather than for concerns about GH excess. Patient one was a 16-year-old who presented with primary amenorrhea and tall stature, and patient two, a 15-year-old, presented with a history of irregular menstruation. Both patients were noted to have acromegalic features, and an extensive work-up confirmed GH-secreting pituitary adenomas. In addition, patient two had significant hyperprolactinemia. Transsphenoidal tumor resection was performed on both patients; patient one had a successful complete resection and achieved endocrine remission, while patient two underwent partial resection followed by a short clinical trial of pegvisomant without significant success. Improved clinical knowledge through case reports can assist with the early diagnosis and management of such rare pediatric conditions.

## Introduction

Gigantism and acromegaly are rare syndromes characterized by excessive growth hormone (GH) secretion, most commonly caused by a pituitary tumor. Growth hormone (GH)-secreting adenomas (somatotropinoma) account for 5%-15% of pediatric pituitary adenomas [1]. Pediatric somatotropinomas tend to be atypical, larger, and more aggressive than those in adults [2], leading to diagnostic and therapeutic challenges in the pediatric population and possibly delaying diagnosis. The clinical presentation in children depends on the status of the epiphyseal growth plates. Before the closure of the growth plates, GH hypersecretion leads to gigantism. The most common and observable clinical sign in children is tall stature [3]. Accelerated growth has been reported at a mean age of 13 years in males, but typically presents earlier in female patients [4]. As the growth plates near complete fusion, the signs, and symptoms in children become more similar to those found in adult patients with acromegaly. These clinical features include acral enlargement, coarse facial features, headaches, and, less frequently, visual changes. Cardiac structural and functional changes such as hypertension, valvular heart disease (aortic and mitral regurgitation), left ventricular hypertrophy, and cardiomyopathy are some of the severe complications seen in both pediatric and adult populations [5]. Somatotropinomas may also be associated with amenorrhea in female patients [6]. A retrospective study of 208 children with pituitary gigantism reported that young age of onset, inadequate GH secretion control, delay in diagnosis, and a high rate of hypopituitarism were associated with significant morbidity [7]. In this study, the pituitary adenomas at diagnosis were already large and, in most cases, had extended and invaded the surrounding structures. Most patients required multimodal treatment, with repeated surgeries, medical therapy, and, in some patients, radiation. In pediatric patients, final adult height is affected by the age of onset, tumor size, and GH level. Attainment of adequate GH secretion control at a younger age minimizes accelerated linear growth and its adverse effect on the final height [7].

We present two patients with pediatric somatotropinomas who were referred to our endocrine practice primarily due to menstrual disorders. Pediatric literature is scarce, and most of what is known about gigantism and acromegaly stems from case reports and guidelines for adult patients. Case reports like ours can guide clinicians in the early diagnosis and management of this rare condition.

## Case presentation

Case one

A 16-year-old female presented to our clinic for evaluation of primary amenorrhea. Puberty began at the age of 12 years. No headaches, vision changes, or galactorrhea were reported. On examination, she was noted to be significantly tall for her age and genetic potential (99.9th percentile for height, +4 SD for age; mid-parental height at the 50th percentile). A review of her growth chart revealed that her height was tracking in the 90th percentile until age 9 years, followed by an accelerated growth velocity of >99th percentile. There had been no previous medical concerns or evaluations for her tall stature. She was obese and had acanthosis nigricans. She was noted to have coarse facial features and large hands and feet. She was at Tanner stage IV for breast development and pubic hair. The bone age was consistent with chronological age and complete growth plate fusion. An ultrasound of the pelvis revealed a prepubescent uterus. Baseline pituitary function labs indicated evidence of GH excess (insulin-like growth factor 1 (IGF-1) 1162 ng/mL (normal level, 108-548)) and possible hypogonadotropic hypogonadism (luteinizing hormone (LH) 0.1 IU/L (0.0-26.4), estradiol 11 pg/ml (2-266)). The rest of her pituitary function was normal (Table 1).

**Table 1:** Baseline laboratory evaluation for patient one

Hormone	Reference range	Result
Growth hormone (random)	0.01-3.61 ng/mL	7.03
Insulin-like growth factor I	108-548 ng/mL	1162
Prolactin	4.8-23.3 ng/mL	8.5
Thyroid-stimulating hormone	0.60-6.39 mIU/L	1.77
Free thyroxine	0.58-1.64 ng/dL	0.86
Luteinizing hormone	0.0-26.4 mIU/ml	0.1 (prepubertal)
Follicle-stimulating hormone	0.4-9.9 (IU)/L	1.6 (prepubertal)
Estradiol	2-266 pg/mL	11 (prepubertal)
Testosterone	0-120 ng/dL	10.01
Free testosterone	1.2-9.9 pg/mL	2.1
Sex hormone-binding globulin	19-145 nmol/L	25

**Figure 1:**
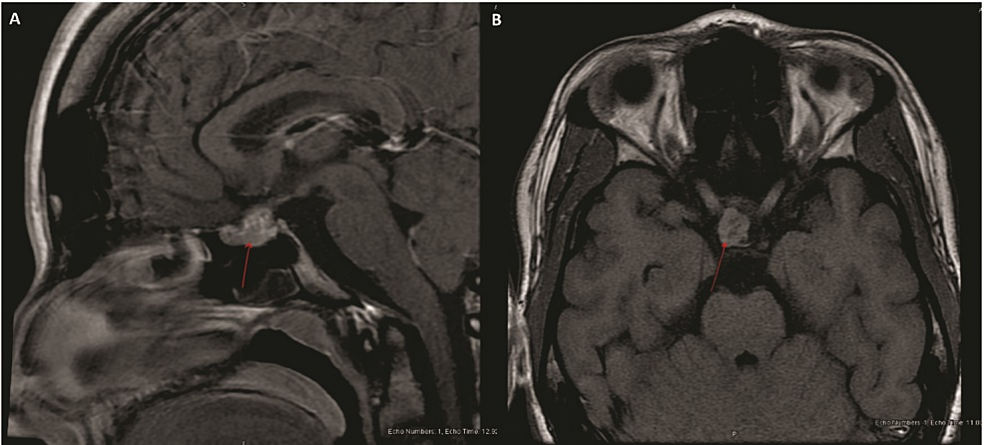
Sagittal and axial T1-weighted images Sagittal T1-weighted image showing a 1.9 x 1.3 x 1.3 cm lobular mass centered in the sella and suprasellar region (A). Axial T1-weighted image showing the mass (B).

Due to these findings, a 75-gram, two-hour oral glucose tolerance test (OGTT), which showed a failure of GH suppression, was completed. The nadir GH level was 4.19 ng/mL (0.01-3.61) at 30 minutes, consistent with GH excess. Magnetic resonance imaging (MRI) of her brain revealed a 1.9 x 1.3 x 1.3 cm lobular mass within the sella and suprasellar regions, with some displacement of the pituitary stalk and optic chiasm (Figure 1).

Her echocardiogram revealed mildly thickened aortic valve leaflets. The visual field exam was normal. She was then referred to neurosurgery and underwent complete surgical removal of the tumor via an endoscopic transsphenoidal approach. On histopathology, the reticulin stains did not show any acinar structures, a pattern that is most characteristic of a pituitary adenoma. Despite normal initial thyroid function tests, her immediate preoperative labs revealed central hypothyroidism, and levothyroxine therapy was started. During the immediate postoperative period, she developed mild and transient diabetes insipidus. Her IGF-1 decreased to 738 ng/mL on postoperative day four and normalized to 373 ng/mL two months later. Several days after surgery, a high-dose adrenocorticotropic hormone stimulation test (using 250 µg cosyntropin intravenously) showed an adequate peak cortisol level of 18.2 mcg/dL. Central hypothyroidism and hypogonadotropic hypogonadism persisted postoperatively, for which she continues to receive treatment. She was subsequently followed as an outpatient and has remained in remission for the past two years without any adjuvant therapy to lower GH levels.

Case two

A 15-year-old female presented to our clinic for evaluation of irregular menstrual cycles. She had a history of normal pubertal development. Menarche was reported at 12 years, and cycles were regular until age 14 years. She also complained of excessive hair growth on her abdomen and rapid weight gain over the previous two years. She had coarse facial features, large hands, and feet, acanthosis nigricans, hirsutism, kyphoscoliosis, a deep voice, and was obese with a body mass index greater than the 99th percentile. Height was at the 50th percentile, with mid-parental height at the 25th percentile. Baseline evaluation of irregular menses indicated a significantly elevated serum prolactin level of 260 ng/mL (3.2-20), low serum luteinizing hormone of 0.14 mIU/mL (0.97-14.70) and an elevated serum free testosterone of 5.6 pg/mL (0.5-3.9). She also had mixed hyperlipidemia, mild hypercalcemia, a serum level of 11.4 mg/dL (normal 8.5-10.4 mg/dL), insulin resistance, and normal thyroid function (Table 2). Repeat labs confirmed hyperprolactinemia (serum prolactin 284.8 ng/mL), and she was started on treatment with cabergoline.

**Table 2:** Baseline laboratory evaluation for patient two

Hormone	Reference range	Result
Insulin-like growth factor I	218-659 ng/mL	928
Prolactin	3.2-20.0 ng/mL	260.5
Thyroid-stimulating hormone	0.50-4.30 mIU/L	0.77
Free thyroxine	0.8-1.4 ng/dL	1.3
Luteinizing hormone	0.97-14.70 mIU/mL	0.14
Follicle-stimulating hormone	0.4-9.9 (IU)/L	0.8
Estradiol	2-266 pg/mL	21
Testosterone	<=40 ng/dL	22
Free testosterone	0.5-3.9 pg/mL	5.6
Sex hormone-binding globulin	12-150 nmol/L	8

**Figure 2:**
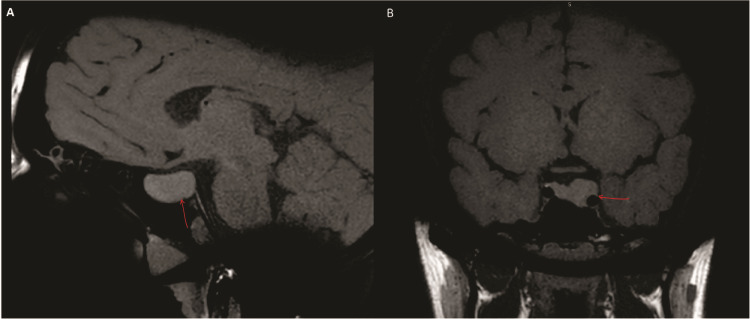
Sagittal and coronal T1-weighted images Sagittal T1-weighted image showing a 1.7 x 1.5 x 2 cm pituitary macroadenoma. The pituitary stalk and remaining pituitary gland are deviated to the right (A). T1-weighted coronal image showing the same macroadenoma invading the cavernous sinus and wrapping around the internal cerebral artery (B).

A brain MRI revealed a pituitary macroadenoma measuring 1.7 x 1.5 x 2 cm, invading the cavernous sinus and wrapping around the internal cerebral artery (Figure 2). Visual field and cardiovascular exams were normal.

Due to these findings, additional pituitary hormonal evaluation was obtained and revealed an elevated IGF-I level of 1374 ng/mL (208-619) and insulin-like growth factor binding protein 3 (IGFBP-3) of 11.2 mg/L (3.4-9.5). A 75-gram, two-hour OGTT showed a failure of GH suppression, with an abnormal GH nadir of 11.7 ng/mL (<10) at 120 minutes. An 8 a.m. serum cortisol level and serum electrolytes were normal except for mild hypercalcemia. She was evaluated by neurosurgery and underwent partial transsphenoidal excision of the macroadenoma due to its critical location. Histopathology was consistent with a pituitary adenoma. Her thyroid function, cortisol, and serum sodium levels remained within normal limits postoperatively. However, IGF-1 and prolactin levels remained elevated over the subsequent two years of follow-up without any adjuvant treatment. She has had recurrent disease and received pegvisomant as part of a clinical trial for 4 months until the family withdrew from therapy. She is being evaluated for possible repeat surgery. Her clinical course was complicated by hypertriglyceridemia (serum triglycerides of 706 mg/dL (normal<90)), which was treated with fenofibrate, and insulin resistance with an elevated hemoglobin A1C level of 6.1%. Additionally, she underwent posterior spinal fusion to correct her kyphoscoliosis.

Informed consent was obtained from the parents or guardians of these patients.

## Discussion

Pituitary gigantism and acromegaly are rare consequences of benign pituitary tumors. Pituitary adenomas are uncommon in children, comprising less than 3% of all supratentorial tumors [7]. Somatotropinomas account for 5% to 15% of pituitary adenomas and are less common than prolactinomas and corticotropinomas [7]. Elevated and uncontrolled serum GH and IGF-1 levels lead to delayed epiphyseal closure, which allows for rapid, excessive linear growth, and if left untreated, extremely tall adult stature ensues [8]. Conversely, acromegaly occurs after the fusion of the epiphyseal plates. The incidence of acromegaly is three to four per million per year and is uncommon in the pediatric population [9]. To date, somatotropin-specific therapeutic guidelines for children do not exist. Therefore, the clinical guidelines for pediatric patients that are typically followed are based on adult care guidelines and pediatric case reports.

The insidious nature of acromegaly causes most cases to be overlooked, which consequently delays the diagnosis [2]. It is also worth noting that a rapid increase in height, shoe, hat, glove, and ring size may be misinterpreted as normal growth and developmental changes, especially in the adolescent population. The limited knowledge of such cases in pediatrics has been emphasized in the literature. Bhattacharjee et al. described a 25-year-old female with a past medical history of tall stature during adolescence and delayed puberty. She experienced frontal headaches for two years before a pituitary macroadenoma diagnosis, and a total of nine years passed from the onset of symptoms until a formal diagnosis was made [10]. This delay in diagnosis often leads to complications as the adenomas become larger and more invasive in approximately 30% to 60% of cases [2]. Both patients described here had macroadenomas at diagnosis. Additionally, the adenoma in patient two invaded the cavernous sinus, which indicated a complicated surgical lesion and a prognostic factor for persistent disease.

Initial common features of acromegaly include coarse facial features, a broadened nose, macrognathia, diastema, and hypertrophy of the hands and feet. These features were present in both of our patients. Other possible clinical manifestations include macroglossia and hypertrophy of pharyngeal and laryngeal tissues, leading to obstructive sleep apnea; hirsutism; hypertrophic arthropathy of the knees, ankles, and spine, leading to joint pain and kyphosis; as well as cardiovascular disease (hypertension, valvular heart disease, cardiomyopathy) [11]. During the preoperative evaluation, patient one was found to have mildly thickened aortic valve leaflets. Patient two’s physical exam was positive for hirsutism and significant kyphoscoliosis, which required surgery.

Noteworthy metabolic effects of acromegaly include amenorrhea or oligomenorrhea, impaired glucose tolerance, insulin resistance, and hypertriglyceridemia [12,13]. Hyperprolactinemia has been noted in approximately 50% of the cases, caused by a somatomammotroph adenoma co-secreting GH or a deviation of the pituitary stalk, causing interference with hypothalamic-pituitary blood flow [14]. Pediatric somatotroph adenomas are usually large, which accounts for the compressive symptoms and local invasion at presentation. In our case report, both patients presented with menstrual disorders as their chief complaint: amenorrhea in patient one and oligomenorrhea in patient two. Both patients were obese and had clinical features of insulin resistance. Patient two presented with 1,25-dihydroxyvitamin D-related hypercalcemia, a direct effect of GH excess, and hyperprolactinemia.

The Endocrine Society’s clinical practice guidelines recommend measuring age-adjusted IGF-1 levels to screen for growth hormone excess [15]. Because of its pulsatile secretion, random GH levels are often unreliable for diagnostic purposes. Biochemical and hormonal assays were used for the initial diagnosis of acromegaly in our patients, revealing elevated age-adjusted IGF-1 levels. This was followed by a 75-gram, two-hour OGTT, which failed to suppress GH levels. Initial evaluation also included analysis of bone age, thyroid function tests, and sex steroid hormone concentrations. Bone age in patient one revealed closed epiphyseal plates. The presence of a pituitary adenoma was confirmed in both patients by brain MRI. In addition to these tests, genetic marker screening tests were obtained to identify germline and somatic mutations known to cause pituitary acromegaly in patient one. Microduplications on chromosome Xq26.3 have been associated with gigantism and acromegaly [16]. The pituitary tumor transforming gene is overexpressed in most somatotroph adenomas and plays a role in tumor invasion of the sphenoid bone [16]. In patient one, these were negative. 

Treatment of somatotropinomas is individualized based on the degree of GH excess and the size and location of the adenoma [17]. Based on the characteristics and behavior of pituitary adenomas, surgery is typically the best course of action [18]. Transsphenoidal resection has shown an excellent prognosis for microadenomas, with 80% to 90% cure rates. The cure rates for macroadenomas, however, are less than 50%. There have been several case reports indicating that pediatric patients have lower success rates and greater postoperative complications. In a retrospective study of 66 pediatric patients (<16 years old) with pituitary adenomas, eight patients with GH-secreting adenomas underwent surgery, which was effective in the minority of patients (12%) [19]. In another study of 44 pediatric patients, surgery alone was successful for 22% of macroadenomas and 44% of microadenomas. Adjuvant therapies, such as secondary surgery, radiotherapy, and/or medical therapy, allowed for the remission of 31% of macroadenomas and 50% of microadenomas [19]. Stereotactic radiation therapy is recommended if there is continued disease after surgery and no improvement with, or intolerance to, other medical modalities. Pediatric somatotropinomas are usually significantly large and invasive, and in some cases, multiple treatment modalities are necessary [15]. Patient two had recurrent disease and was briefly treated with pegvisomant with consideration for secondary surgery.

## Conclusions

Rapid growth in adolescents with excess growth hormone may be misinterpreted as a pubertal growth spurt. Acromegaly in adolescents may present with symptoms not directly related to growth hormone excess, such as menstrual irregularities in our patients. All these factors may delay the diagnosis. Partly due to these reasons, pediatric pituitary adenomas tend to be atypical, larger, and more aggressive than those in adults. Pediatricians and endocrinologists need to consider growth hormone excess in children presenting with other pituitary hormone-related symptoms if the patient also has or has had subtle signs of growth hormone excess. Improved patient care in such rare conditions is achievable with better knowledge and coordinated care by a multidisciplinary team of pediatricians, endocrinologists, neurosurgeons, and otolaryngologists.
